# Hole Polaronic Confinement in (111) Yttria‐Stabilised Zirconia

**DOI:** 10.1002/smll.202513940

**Published:** 2026-03-12

**Authors:** Milica Vasiljevic, Victor Buratto Tinti, Javier Zamudio‐García, José Maria Castillo Robles, Vasileios Bilalis, Imran Asghar, Simone Santucci, Yichen Wu, Simone Sanna, Carmela Aruta, Pasquale Orgiani, Dimitrios Koukoulis, David Marrero‐López, Weimin Wang, Ivano E. Castelli, Vincenzo Esposito

**Affiliations:** ^1^ DTU Energy Technical University of Denmark Lyngby Denmark; ^2^ Renewable Energy Technologies Group Faculty of Engineering and Natural Sciences Tampere University Tampere Finland; ^3^ Universita' Degli Studi Di Roma Tor Vergata Department of Civil Engineering and Computer Science Roma Italy; ^4^ CNR‐SPIN Roma Italy; ^5^ CNR‐IOM Trieste Italy; ^6^ Universidad de Málaga Dpto. de Física Aplicada I Málaga Spain; ^7^ Unversity of Lund MAX IV Science Division Lund Sweden

**Keywords:** density functional theory (DFT), non‐classical electrostriction, nanoionics, nanostructures, quantum‐localised defects, small polarons, thin films, yttria‐stabilised zirconia (YSZ)

## Abstract

Yttria‐stabilized zirconia (YSZ) is the benchmark oxygen‐ion conductor and is widely regarded as electronically inert under oxidizing conditions. Yet its electrical behavior at the nanoscale remains unsettled. While bulk YSZ exhibits predominantly ionic transport, electronic contributions have only been reported under highly defective, porous, or strong‐field conditions. Here, we demonstrate that ultrathin epitaxial YSZ films (<20 nm) exhibit measurable p‐type mixed conduction at room temperature arising intrinsically from crystallographically ordered defect–dopant associations. Combined electrical measurements and first‐principles modeling show that Y^3^^+^–vacancy complexes stabilize hole polarons confined along specific lattice directions. In (111)‐oriented films, interfacial defect ordering produces a high density of confined polarons, enabling directional charge transport and enhanced electro‐chemo‐mechanical coupling beyond classical electrostriction. These results show that electronic functionality in YSZ can emerge solely from nanoscale defect ordering, redefining its transport behavior beyond the classical purely ionic paradigm and revealing unexpected electromechanical functionality in a canonical ionic oxide.

## Graphical abstract

Ultrathin epitaxial YSZ(111) films unexpectedly develop p‐type mixed conduction and amplified electromechanical coupling under oxidizing conditions. Combined transport measurements and first‐principles modeling reveal that nanoscale confinement and ordered dopant–vacancy complexes stabilize localized O 2p hole polarons at the surface. A canonical ionic conductor thus acquires orientation‐controlled electronic functionality, redefining defect engineering in nanoionic oxides.

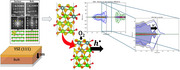

## 1 Introduction

Yttria‐stabilized zirconia (YSZ) remains the benchmark oxygen‐ion conductor and a foundational material in solid‐state electrochemical devices, including fuel cells, oxygen sensors, and oxygen‐permeable membranes [1, 2, 3, 4, 5, 6, 7]. Its high chemical stability and refractory nature support oxygen‐vacancy conduction of approximately 10^−3^ S cm^−1^ at 600°C, with an activation energy near 1 eV [8, 9]. The host cation Zr^4+^ maintains a fixed valence over broad ranges of temperature and oxygen partial pressure, ensuring predominantly oxygen‐vacancy ($V_{O}^{\cdot \cdot }$) mediated ionic transport [10, 11].

Electronic conductivity is well established in reducible fluorite oxides such as ceria, where mixed ionic–electronic conduction readily emerges under moderate reducing conditions [12, 13]. In contrast, bulk YSZ under oxidizing environments is widely regarded as a purely ionic conductor, with electronic contributions that are small and strongly condition‐dependent.

Partial electronic conductivity in YSZ has nevertheless been reported under specific regimes. Porous YSZ films exhibit enhanced electronic partial conductivity attributed to surface space‐charge effects [14]. Bias‐induced electronic conduction has been observed in nanometer‐thick YSZ films under applied DC fields [15], and field‐driven p‐type‐to‐n‐type transitions under strong polarization [16]. Mixed electronic–ionic behavior also arises in engineered YSZ–graphene composites once a percolating electronic network forms [17, 18]. These cases reflect non‐equilibrium or architecture‐dependent effects rather than intrinsic mixed conduction in dense bulk YSZ.

Under strongly reducing conditions (pO_2_< 10^−^^1^ ^7^ atm, T > 900°C), n‐type electronic conductivity in YSZ can approach ∼10^−^^5^ S cm^−^^1^ at high temperature due to electron carrier formation [19, 20]. In air, the electronic contribution remains several orders of magnitude lower than the ionic conductivity and typically falls below ∼10^−^^6^ S cm^−^^1^ at intermediate temperatures [19]. Even small electronic leakage can influence device efficiency and nanoionic functionality [21, 22, 23], yet the microscopic origin of mixed conduction in YSZ remains debated. While often attributed to electron polarons associated with oxygen‐vacancy formation and recombination, such mechanisms are energetically demanding in stoichiometric zirconia and may not fully account for reported observations.

Mixed conduction in YSZ is often associated with extrinsic factors such as aliovalent dopants, impurities, interstitial defects, charged grain boundaries, or metastable phases [24]. At the nanoscale, defect chemistry and transport can be modified by charged interfaces and strain fields that alter vacancy mobility and carrier localization [11, 12, 13, 25]. Nanostructuring in YSZ has primarily been linked to enhanced ionic conductivity via interface‐mediated effects rather than dominant electronic transport [26, 27, 28]. Apparent p‐type behavior in YSZ/STO multilayers has been attributed to the STO interlayers rather than YSZ itself [29].

In contrast to n‐type conduction under strongly reducing conditions, p‐type conductivity in YSZ is especially rare under equilibrium oxidizing environments. When detected, it is typically associated with non‐equilibrium driving forces such as an applied DC bias, in which hole conduction has been linked to O‐related defect species and surface redox equilibria [15]. Given the strong dopant–vacancy association in acceptor‐stabilized zirconia, intrinsic vacancy annihilation is not energetically favored [30], and reported p‐type contributions are therefore often attributed to surface, interfacial, or extrinsic defect effects.

Beyond classical defect equilibria, oxide defects can host localized, lattice‐coupled electronic states consistent with polaron formation [31]. Such defect–lattice coupling provides a physically consistent microscopic framework for understanding electronic contributions in otherwise ionic fluorite oxides. Whether nanoscale confinement and interfacial defect ordering alone can stabilize measurable p‐type conduction in epitaxial YSZ under oxidizing conditions remains unresolved.

Here, we investigate the electronic behavior of YSZ nanoionic thin films with controlled orientation and interface structure. We demonstrate that nanoscale confinement stabilizes small‐hole polarons, leading to measurable p‐type conductivity and electro‐chemo‐mechanical coupling even at room temperature in an oxide traditionally regarded as purely ionic. These findings show how controlled defect–lattice coupling can endow a classical ionic conductor with emergent electronic and electromechanical functionality.

## 2 Results and Discussion

To highlight the effects at the nanoscale, we use oriented epitaxial thin films, i.e., coherent and microstructurally homogeneous, along selected crystallographic orientations. YSZ films with well‐defined thicknesses, including thin (nominally 20 nm, measured 15–20 nm), intermediate (50–70 nm), and thick (250 nm), as well as different crystal orientations, were grown on single‐crystal substrates using pulsed laser deposition (PLD). Although lattice strain is expected to persist only within a few nanometers of the substrate interface, variations in film thickness can influence strain relaxation and the resulting electrical properties in the coherent film. On the other hand, for nominal lattice mismatches above 5–6%, deposition generally results in semi‐coherent films with vertically aligned domains and grain boundaries, i.e., columnar growth. By carefully selecting substrates and PLD deposition parameters, we ensure the growth of coherent or semi‐coherent YSZ films, depending on thickness, with low mosaic spread and high crystalline quality. Additionally, the substrate single crystal's orientation results in different orientations in the resulting thin films.

**FIGURE 1:**
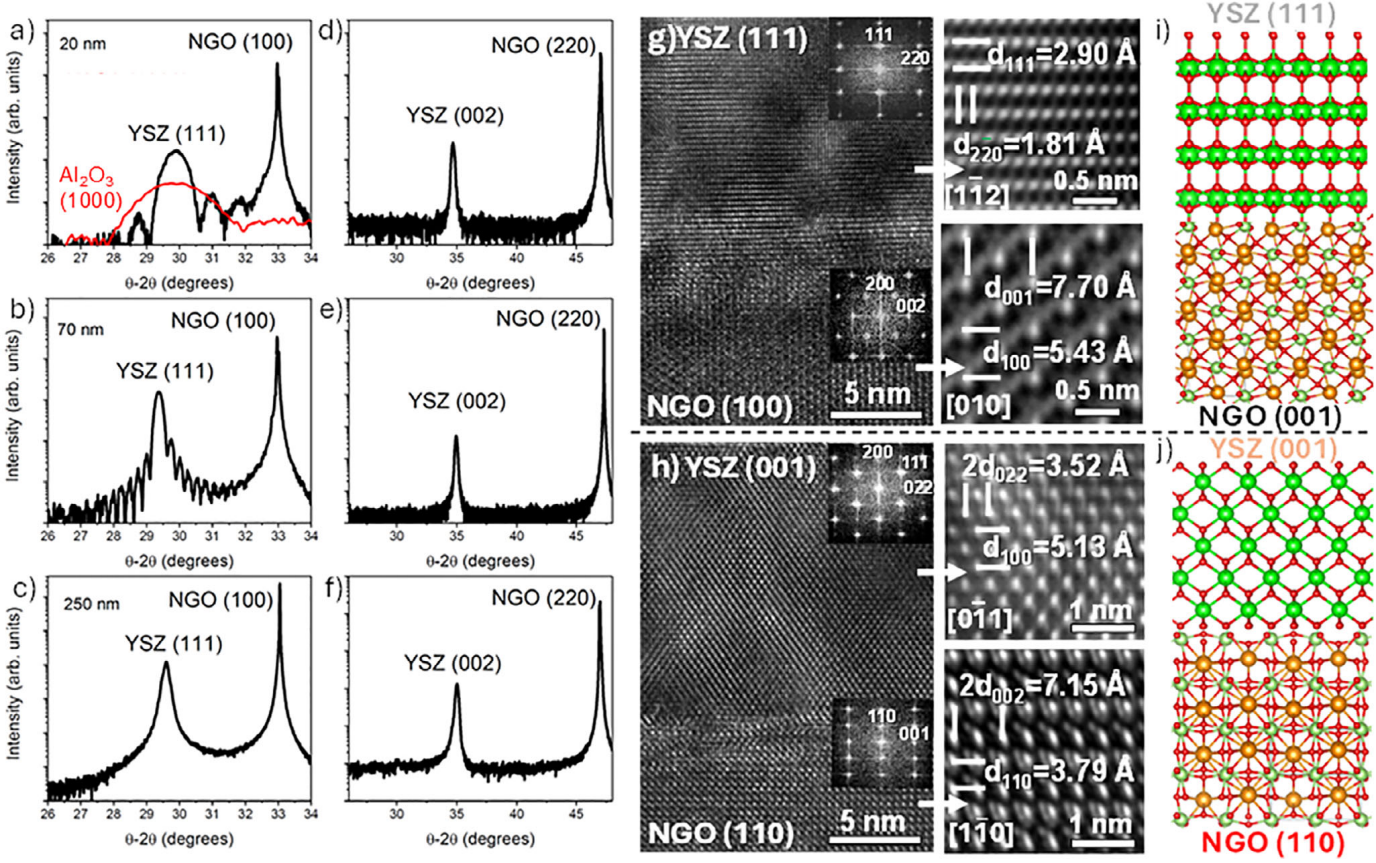
XRD patterns of YSZ (111) films on NGO (100) substrate with different thicknesses of (a) 20 nm, (b) 70 nm, and (c) 250 nm. XRD patterns of YSZ (111) films on NGO (100) substrate with different thicknesses: (d) 20 nm, (e) 70 nm, and (f) 250 nm. Cross‐sectional HR‐TEM images showing the epitaxial growth of (g) YSZ (111) and (h) YSZ (001). Schematic illustrations showing the atomic arrangement of (i) YSZ (111) and (j) YSZ (001).

Figure 1 shows the structural features and schematics of the thin (20 nm), intermediate (70 nm), and thick (250 nm) YSZ films deposited on NdGaO_3_ (NGO) with (100) and (110) orientation, respectively. The X‐ray reflectivity (XRR) measurements confirm the film thicknesses (see Figure S1a,b).

All the YSZ films on NGO (100) are oriented along the (111) direction (Figure 1a–c), while YSZ on NGO (110) exhibit a preferential growth along the (100) direction (unique reflection at (200)), regardless of their thickness, as shown in Figure 1d–f (YSZ on NGO (110)) and Figure S1c (corresponding RSM). The nominal lattice mismatch between the YSZ and NGO lattices (∼5%) is expected to promote structural dislocations^4^ ^1^ at the film/substrate interface. Consistent with this, XRD measurements reveal peak shifts on the order of ∼1° in 2θ for thinner samples (Figure 1a–c), indicating significant compressive strain. However, these shifts plateau in thicker films, suggesting partial strain relaxation with increasing thickness. While no long‐range strain gradient is evident across thicknesses, local interface strain and crystallographic orientation may still play a decisive role in the electronic behavior of the thinner samples.

The atomic arrangement is investigated using HR‐TEM and Energy Dispersive X‐ray (EDX) spectroscopy on cross‐sectional thin lamellas of samples deposited on NGO (100) and (110) (Figure 1i,j; Figures S2 and S3). YSZ films on NGO (100) exhibit high‐quality epitaxy with coherent growth maintained in the thinner films, oriented along the (111) plane. In contrast, on NGO (110), a preferential growth along (100) is observed, with no appreciable crystallographic misorientations within the resolution of our measurements, as previously confirmed by XRD (Figure 1a,b) and reciprocal space map (RSM, Figure S1c).

Fast Fourier Transform (FFT) analysis further validates the high coherency of both epitaxial films, revealing in‐plane lattice matching relationships between NGO (100) || YSZ (220) and NGO (110) || YSZ (220), as well as out‐of‐plane matching relationships between NGO (100) || YSZ (111) and NGO (110) || YSZ (001). The interplanar distances measured by HR‐TEM match those obtained by XRD and are consistent with theoretical values, confirming the epitaxial growth. Figure 1i,j schematically illustrates the atomic arrangement of YSZ on the different substrates.

Although bulk fluorite YSZ is cubic and macroscopically isotropic, defect segregation and electronic localization can become strongly orientation‐dependent at interfaces and surfaces, where symmetry is locally broken. Therefore, the anisotropies of the ionic and electronic defect configurations in the distinct (100) and (111) growth orientations are expected to influence charge carrier distributions and mobility, particularly in the nanoscale regime. Moreover, these structural characterizations confirm the precise control of crystallographic orientation and coherence across thicknesses, laying the foundation for understanding the emergent electronic behavior in the following sections.

For the electro‐chemo‐mechanical properties, we characterize the films at high and low temperatures. Impedance spectroscopy is used to investigate the effects of film orientation and thickness on electrochemical properties. We measure the in‐plane conductivity of the samples (σ_p_) as a function of the temperature and oxygen partial pressure using lateral silver electrodes on the films. First, an electrical characterization of the bare substrates is performed to confirm their insulating nature, with electrical conductivity << 10^−4^ S cm^−1^ at 700°C in any pO_2_ conditions. These results are consistent with previous measurements using a 2‐probe in‐plane electrode configuration under similar conditions and substrates [32].

**FIGURE 2:**
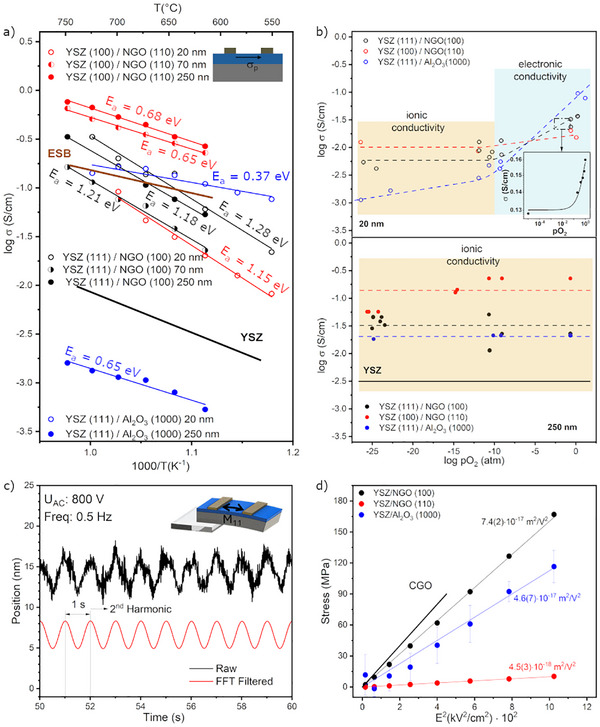
(a) Arrhenius plot of conductivity of various YSZ thin films with different thicknesses and a schematic of the in‐plane conductivity measurement of the samples as an inset. (b) Brower plot for YSZ thin (up) and thick films (down). (c) Raw and processed data of the second‐order electromechanical response with an inset of the experimental setup's schematic and sample configuration. The samples were arranged in a cantilever geometry, and the electric field was applied to the in‐plane electrodes. (d) Electromechanical response under an alternating electric field at 5 Hz measured at 10 Hz (second Harmonic) for the 20 nm thick films. All values reported are absolute. Error bars for both YSZ samples deposited on NGO are smaller than the symbol size and are therefore not visible in the graph; detailed values are provided in Tables S1 and S2.

The Arrhenius plot in Figure 2a is used to analyze conductivity as a function of temperature. The study is conducted in air, i.e., the oxygen partial pressure is kept constant at 0.21 atm. Epitaxial YSZ/NGO thin films of 20 nm and above show higher conductivity than the reference YSZ bulk samples, with maximum conductivity (>0.5 S cm^−1^ at 700°C) observed in thick YSZ (100). Typical Nyquist plots with equivalent circuits are reported in Figure S4.

This performance is comparable to that of fast conductors, e.g., Er‐Bi_2_O_3_ (ESB) and other nanostructured oxygen ionic conductors [33, 34]. However, the conductivity of YSZ (100) decreases with decreasing film thickness, falling below that of YSZ (111). In contrast, the conductivity of YSZ (111) films increases with decreasing thickness. Moreover, while the 1.2 eV activation energy for YSZ (111) remains nearly constant at thicknesses between 20 and 250 nm, a thickness dependence of activation energy for YSZ (100) is observed. Thin (20 nm) YSZ (100) films have the same activation energy as bulk films, while thicker films show a reduction to 0.7 eV, similar to the bulk YSZ [19].

To investigate the conductivity dependence on the film orientation and thickness, we measured an additional sample on sapphire, i.e., YSZ (111)/Al_2_O_3_ (1000). For these samples, the lattice mismatch is significant (∼7%), and a columnar‐like morphology with a preferential (111) orientation forms [35]. This structural feature results in a wider FWHM of the YSZ (111) diffraction peak, as shown in Figure 1a, and a schematic of the YSZ thin film on sapphire is shown in Figure S5. The YSZ/Al_2_O_3_ exhibits a lower activation energy. However, conductivity changes abruptly, decreasing by two orders of magnitude when the thickness is reduced from 250 to 20 nm. In the ultrathin YSZ/Al_2_O_3_ case, the large mismatch promotes a defective semi‐coherent interface that likely dominates the measured transport response. The observed p‐type contribution is therefore attributed primarily to interfacial confinement rather than bulk YSZ conduction.

In summary, the Arrhenius plots show that the sample's orientation and thickness influence the activation energy. The relatively wide variations in the activation energies and conductivities measured in the samples suggest that multiple transport mechanisms are at work. These results reflect the properties’ changes previously reported in zirconia [4, 5, 11, 36] and highlight that electrical transport instability occurs at the nanoscale.

To investigate the nature of conductivity in YSZ samples, we performed impedance measurements as a function of oxygen partial pressure (pO_2_), presented as Brouwer‐like plots in Figure 2b. The temperature was maintained at a constant value (T = 625°C) during the measurements, while the oxygen partial pressure was varied from 10^−25^ to 1 atm. The typical Brouwer‐like plot for a pure ionic conductor shows a constant value over the entire pO_2_ range. This characteristic is typically observed for the YSZ bulk sample at T = 625°C and under measurement conditions of 10–25< pO_2_< 1 atm. Conversely, for mixed ionic‐electronic conductors (MIECs), conductivity is a function of pO_2_ [34].

Notably, while all thick films exhibit purely ionic conductivity, some thin films display p‐type electronic conductivity behavior, particularly pronounced in YSZ (111) (Figure 2b up). To further illustrate this, we include an inset showing measurements performed with blocking electrodes in the range 1 ≥ pO_2_ ≥ 10^−^^5^ atm (see Figure S6), plotted on a linear scale to emphasize the smoother, more distinct p‐type slope. Although this effect is measurable and spans 3‐4 orders of magnitude, a consistent slope in the high‐oxygen regime (pO_2_> 10‐10 atm) is not observed, indicating instability in the electrochemical mechanism. The polaronic contribution is observed only in thin films (see Figure 2b). It was absent in thicker films, suggesting that the electronic contribution is primarily interfacial and does not represent the overall transport process. Consequently, the results reflect the behavior of a nanometric portion of the thin film rather than the entire material.

The presence of quasi‐free electrons at the surface has significant implications for O_2_ gas‐solid exchange, defect formation and annihilation mechanisms, and practical applications in high‐temperature electrodes, such as gas sensors, solid oxide fuel cells, and electrolyzers [37, 38, 39, 40]. Furthermore, recent studies have shown that point and electronic defects can induce high electromechanical coupling in fluorite‐based ceramics at room temperature [23, 41, 42].

With this in mind, we analyze the electromechanical response of 20 nm thin films deposited on NGO and Al_2_O_3_ at room temperature. The samples are arranged in a cantilever geometry, and the electric field is applied to the in‐plane electrodes; see Methods and Figure S7a for detailed information. Schematics of the experimental setup and sample configuration are shown as an inset of Figure 2c and Figure S7, together with the raw and processed data of the second‐order electromechanical response. Interestingly, all samples exhibit a second‐order reaction that is linear to the square of the applied electric field (Figure 2d), consistent with electrostrictive behavior. The YSZ/NGO (110) film exhibits an electrostrictive coefficient in the lower range (M_11_ ≈ 10^−18^ m^2^V^−2^) of the “non‐classical” electrostrictive behavior [43].

The films deposited on NGO (100) and sapphire show a significantly higher *M_11_*, approximately one order of magnitude higher (M_11_ ≈ 10^−17^ m^2^V^−2^) than the sample deposited on NGO (110), comparable to the enhanced electrostriction measured in Gd‐doped CeO_2_. The samples with enhanced electromechanical responses are the same ones that exhibit MIEC features. These results align with the previous work, indicating that polaronic features enhance the electromechanical response [23, 41].

**FIGURE 3:**
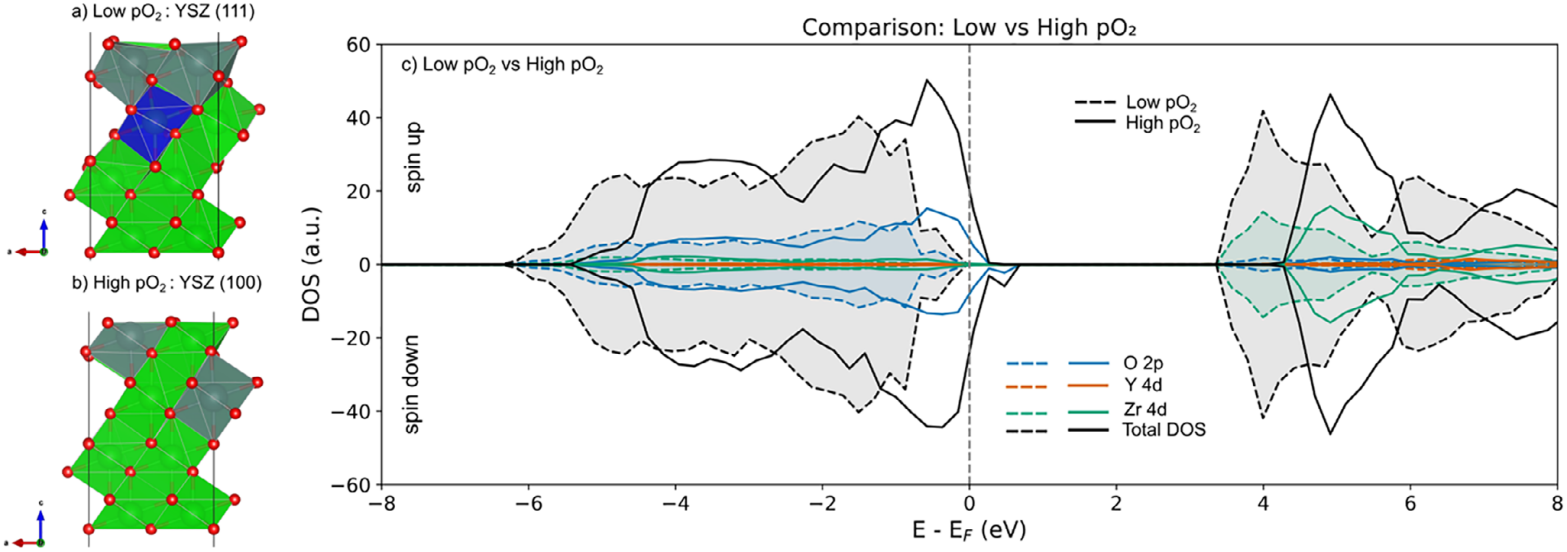
(a) Schematic representation of the YSZ structures used in the DFT calculations, illustrating the most favorable positions for Y‐doping and oxygen vacancies (Vö) in (a) YSZ (111) and (b) YSZ (100) thin films. The YSZ (111) surface, representative of low pO_2_ conditions, contains surface oxygen vacancies, whereas the YSZ (100) surface, corresponding to high pO_2_ conditions, lacks surface vacancies. Green and gray polyhedra represent Zr and Y atoms, respectively, while blue polyhedra highlight Zr sites associated with oxygen vacancies. Red spheres denote oxygen atoms. (c) Density of states (DOS) calculated for both pO_2_ scenarios, with the Fermi level (E_F_) aligned at 0 eV. Dotted and solid lines correspond to the DOS of low and high pO_2_ conditions, respectively. For visual clarity, the area under the DOS curve of the low pO_2_ case is shaded to highlight the electronic states.

Given the compelling experimental evidence for MIEC in YSZ (111) thin films, ab initio calculations were performed to provide atomistic insight into the effects of crystallographic orientation and oxygen partial pressure on charge‐carrier behavior in YSZ films. Two orientations, YSZ (100) and (111), were studied and are shown in Figure 3a. The results indicate that crystallographic orientation influences the segregation of oxygen vacancies and dopants, thereby affecting the electronic structure. For YSZ (111), oxygen vacancies preferentially segregate to the surface under low pO_2_ conditions, while YSZ (100) remains more stoichiometric. This is reflected in the density of states (DOS) (Figure 3c), where the low pO_2_ case shows O 2p states at the edge of the Fermi level. When oxygen vacancies are filled (simulating high pO_2_), these O 2p states increase in intensity and begin to cross the Fermi level. The presence of O 2p states at the Fermi level suggests the emergence of hole‐like states and supports the possibility of p‐type conductivity. To complement this analysis, the partial electronic charge density is shown in Figure S8 for the high pO_2_ case, revealing that the electronic density is localized on surface oxygen atoms. The partial electronic density was integrated over 0–0.5 eV relative to the Fermi level. The DOS for the high pO_2_ configuration shows that the Fermi level is localized at the top of the O 2p valence band, with a small DOS at the Fermi level, reflecting a localized partial electronic density, as shown in Figure S8, rather than delocalized metallic Bloch states.

This identification is substantiated by a Bader charge analysis, which assigns a charge of approximately ‐1 to the corresponding oxygen atom, consistent with the formation of an O^−^ hole state. The formation of such a hole polaron is consistent with previous reports for materials such as Yttria‐stabilized zirconia (YSZ), where hole polaronic states have been demonstrated even in vacancy‐free structures [6]. A notable distinction, however, arises from comparison with these prior studies, which utilized bulk supercell models and reported two localized states corresponding to (O_2_)^3−^ complexes. The observation of a single localized polaron in the present work is therefore attributed to the surface configuration, which provides a unique electrostatic environment for charge localization that is absent in the bulk.

**FIGURE 4:**
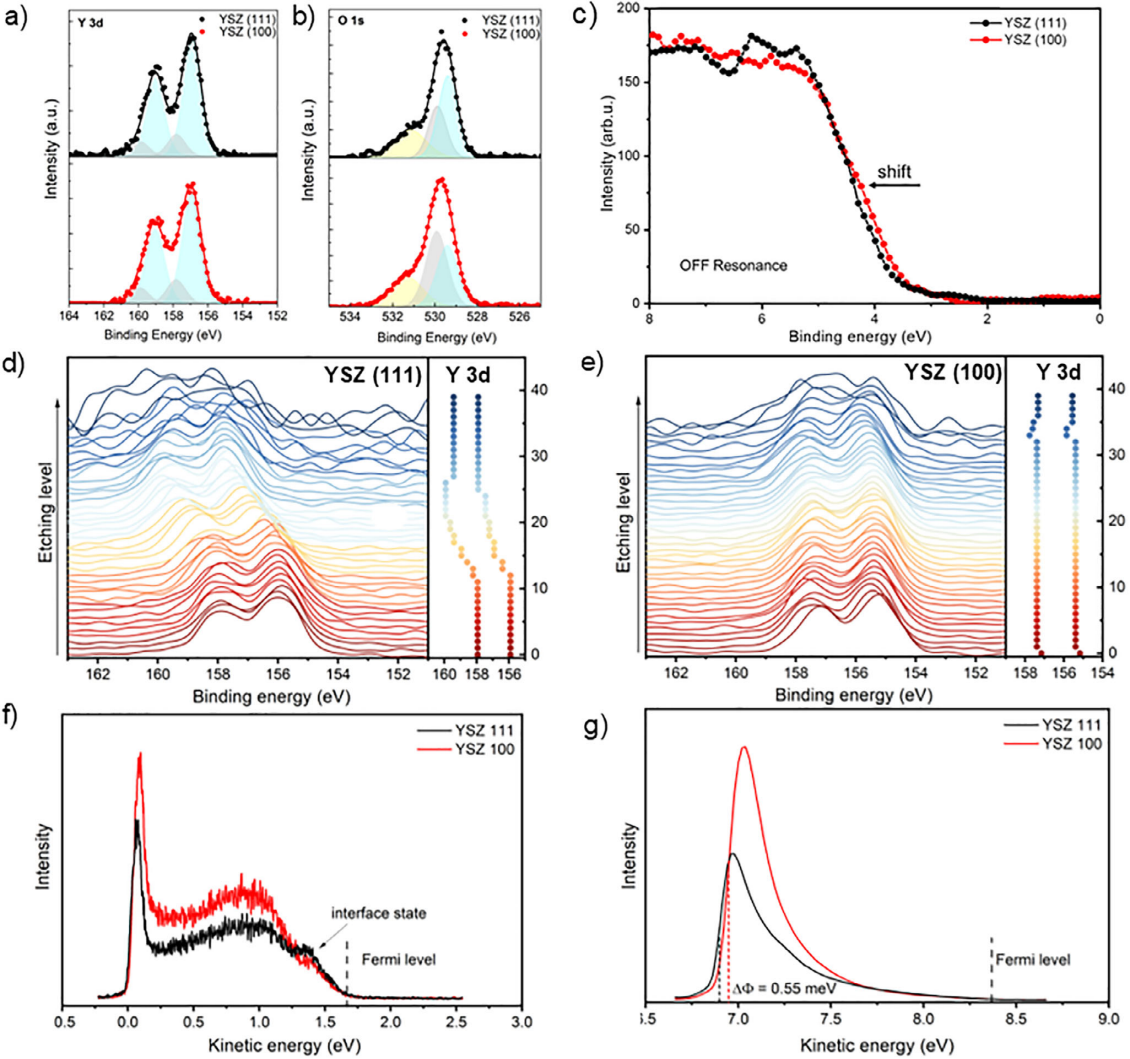
In situ XPS spectra of YSZ (111) and YSZ (100) films on NGO (100) and NGO (110), respectively, corresponding to (a) Y 3d, (b) O 1s core levels and (c) VB measurements at 800 eV excitation energy after normalization at 10 eV. In‐depth XPS spectra of Y 3d (right panels) and highlighted shift of Y 3d peaks (left panels) throughout ion milling in (d) YSZ (111) and (e) YSZ (100) thin films. Ultraviolet photoelectron spectroscopy (UPS) spectra of YSZ (111) and YSZ (100): (f) without and (g) with bias. The unbiased UPS was measured by 6 eV photon energy. The biased UPS was measured by 5.5 eV photon energy. The work function difference (ΔΦ) between the samples was determined from biased UPS, revealing that YSZ (111) has a 55 meV lower work function than YSZ (100).

Several experimental methods confirm the calculations and experimental evidence of polaronic segregation in thin YSZ (111) films. Surface‐sensitive XPS measurements were performed in situ by synchrotron radiation for YSZ (111) and YSZ (100) films deposited on NGO single crystals with intermediate (50 nm) thickness. Y 3d and O 1s core levels plots are reported in Figure 4a,b, valence band (VB) in Figure 4c, while Zr 3d is presented in Figure S9a.

The fit performed on the Y 3d core levels (Figure 4a) reveals two spin‐orbit doublets, 3d5/2 and 3d3/2. According to the literature [44], the doublet at lower binding energy (about 157.0 eV) corresponds to reduced Y ions. The doublet at higher binding energy (about 157.8 eV) corresponds to the stoichiometric chemical environment. The reduced contribution is considerably more pronounced than the stoichiometric one in both Y 3d spectra, but is more pronounced for the YSZ (111) film, thus demonstrating a more considerable content of oxygen vacancies. This effect is even more evident in the O 1s spectra shown in Figure 4b. Three components, corresponding to three different chemical environments of O ions, were used to fit the spectra. The one at the highest binding energy (about 531.3 eV, yellow peak) is assigned to the Zr‐O pair, and the one in the middle (about 530.0 eV, grey peak) to Y‐O. In comparison, the component at the lowest binding energy (about 529.4 eV, light blue peak) is associated with $V_{O}^{\cdot \cdot }$‐Y‐O [45]. The fitting results for the latter component indicate that the concentration of oxygen vacancies is higher on the surface of YSZ (111) than in YSZ (100), further confirming the modelling results. Finally, the Zr 3d core‐level components (Figure S9a), corresponding to the 3d5/2 and 3d3/2 spin‐orbit splitting, do not show substantial differences at the sample surface.

To complement the XPS analysis, we perform resonant photoemission electron spectroscopy (ResPES) on thin YSZ(111) (Figure S9b,c). We use conductive Nb‐doped SrTiO3 (STN) substrates to avoid surface charging during the measurements. However, the orientation and quality of the films remain unchanged (see Figure S10). In Figure S9b,c, we report valence‐band (VB) results, normalized by area, for the YSZ (111) film at different photon energies, spanning the XAS Zr M3 edge. In the ResPES results, no localized states emerge due to oxygen vacancies associated with Zr ions in the gap when the excitation energy varies across the XAS spectrum of the Zr M_3_‐edge. It confirms that the 4d levels of Zr^4+^ are empty, and the valence band (occupied states) is mainly composed of O 2p orbitals, although hybridized with Zr 4d and Y 4d bands, as indicated by the model. When the excitation energy corresponds to the maximum of the Zr M_3_‐edge, the Zr yield increase is observed at the leading edge of the VB, as highlighted in Figure S9b.

Moreover, the XAS spectra in Figure S9b show the double features at the absorption maximum, which is typical for zirconia with a tetragonal structure [46]. Finally, in Figure 4c, we compare the VB spectra of YSZ (100) and YSZ (111) films far from the Zr M‐edge after normalization at 10 eV. Unfilled vacancies in oxygen sites create localized donor vacancy states ($V_{O}^{\cdot \cdot }$‐O) close to the conduction‐band minimum [47, 48]. Therefore, the Fermi level, fixed at 0 eV, shifts closer to the conduction band and farther from the valence band, as predicted by modelling in Figure 3c. Consequently, the shift farther from the Fermi level observed for YSZ (111) indicates a larger concentration of oxygen vacancies (Figure 4c). Because the hole‐polaron states are highly localized and confined to the topmost layers, their spectral weight in VB XPS is expected to be weak, explaining the absence of a distinct feature at the valence edge.

As the model in Figure 3c suggests, the dopant could promote oxygen vacancy segregation and p‐type polaronic confinement. Therefore, we performed in‐depth XPS on YSZ (111) and YSZ (100) thin films to complete the analysis of our samples. The binding energy of Y in YSZ (111), Figure 4d, is shifted toward higher values compared to YSZ (100) in Figure 4e. This increase points to different chemical states of Y relative to the surface; deeper layers contain more oxidized Y species (e.g., Y^3^^+^), leading to an increase in binding energy due to a more substantial effective nuclear charge acting on the 3d electrons [49]. Moreover, as we probe deeper into the film thickness, there is a shift toward higher binding energies of the core levels in YSZ (111). This effect indicates changes in chemical states or coordination environments, revealing that the surface is reconstructed compared to the interface between YSZ (111) and NGO (100). Typically, oxygen vacancies in YSZ lead to lower binding energies for Zr and O, as shown in Figure S10. These vacancies create a more reduced local environment, effectively increasing electron density around the Zr and O sites. On the contrary, if the binding energies shift higher, as seen at deeper etching levels (Figure S10), the bonding environment around Zr and O becomes stronger and more stable. This stability often arises when there are fewer oxygen vacancies, as more complete coordination can enhance the effective nuclear charge felt by the electrons [49]. Moreover, the change in atomic concentration through the samples is more pronounced in the YSZ (111) than in (100) oriented samples, as presented in Figure S11. The chemical inhomogeneity for the thin YSZ (100) film on NGO (110) is observed only in the first few interfacial layers, as indicated in Figure 4e. In contrast, the thicker YSZ samples exhibit no substantial chemical shifts or compositional gradients in the in situ XPS measurements, indicating a largely homogeneous chemical environment throughout the film thickness (see Figure S12).

*Ex situ* ultraviolet photoelectron spectroscopy (UPS) measurements were conducted on very thin YSZ (111) and (100) oriented samples deposited on STN, see Figure S13, to investigate their electronic structures. According to the universal curve of the inelastic mean free path (IMFP) for excited photoelectrons, when using a photon energy of 6 eV, the kinetic energy of valence electrons falls within the range of ∼0 to 1.5 eV, corresponding to an extremely long IMFP (>10 nm), see Figure 4f. However, at such low energies, the valence‐electron signal overlaps with the secondary‐electron (SE) background from inelastic scattering, making it difficult to accurately determine the SE cutoff energy. To solve this issue, a constant bias voltage of −7.23 V was applied to the sample, shifting the valence electrons to higher kinetic energies, as shown in Figure 4g. UPS spectra acquired from multiple positions on the samples showed slight variations. Notably, all spectra from the YSZ (100) showed higher SE intensity (peaking near 0 eV kinetic energy in unbiased measurements), indicating lower conductivity. Because electrons in higher conductive materials lose energy more efficiently to the lattice before escaping, thereby suppressing SE emission. The work function difference (ΔΦ) between the samples determined from biased UPS, revealing that YSZ (111) has a 55 meV lower work function than YSZ (100), as predicted by DFT.

**FIGURE 5:**
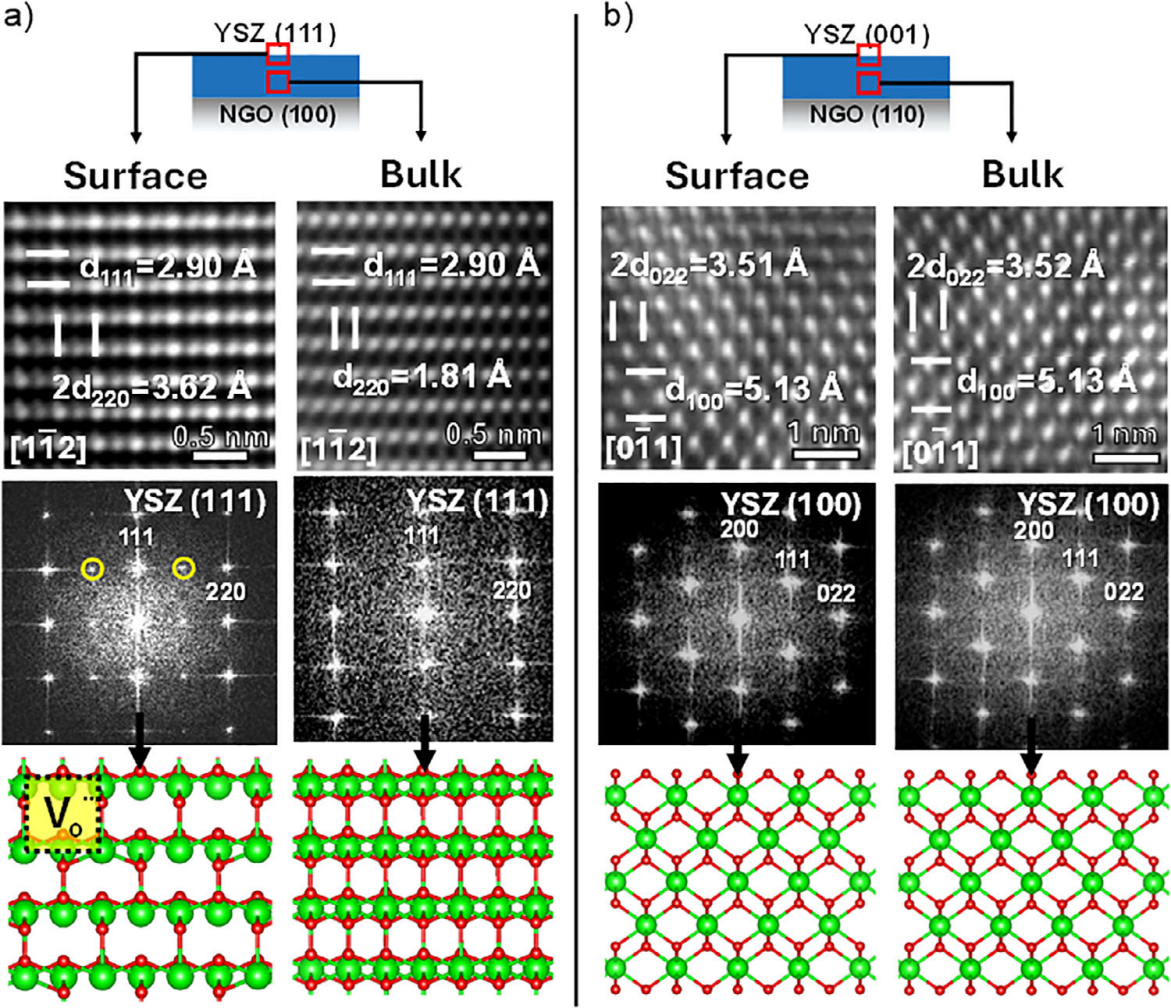
HR‐TEM images of: (a) YSZ (111) close to the surface (left) and within the bulk (right); and (b) YSZ (100) close to the surface (left) and in the bulk (right) of the film, revealing the formation of an atomic ordering along the (220) direction.

The HR‐TEM analysis also confirms the DFT and XPS results at the atomic level. Figure 5 compares the HR‐TEM image of the YSZ (111) and the YSZ (100) samples near the surface and the core film. The YSZ (111) sample shows the reconstruction, as seen in Figure 5a, while the YSZ (100) does not, as shown in Figure 5b. Consistent with the model, a stacking effect is observed at the surface and the substrate interface for YSZ (111). Specifically, the diffraction pattern in the left part of Figure 5a shows additional reflections in the (220) direction for the YSZ (111) film, indicating a sublattice at the film surface, similar to a 2 × 2 superstructure typical of defective fluorites with a high concentration of oxygen vacancies [50, 51]. Notably, the YSZ (100) does not exhibit these features, ruling out artefacts introduced during sample preparation.

## 3 Conclusions

This work demonstrates that electronic defects in YSZ thin films acquire distinct spatial and electronic configurations at the nanoscale, redefining the conventional view of zirconia as a purely ionic conductor. By correlating crystallographic orientation, defect ordering, and transport behavior across coherently grown (100) and (111) films, we establish a direct link between structural symmetry and electronic localization.

Our combined experiments and first‐principles modeling reveal that Y^3+^ dopant segregation promotes the formation of ordered oxygen‐vacancy arrangements, which stabilize hole‐like polarons in near‐surface regions. These confined O^−^ states behave as quantum‐localized charge centers strongly coupled to lattice vibrations, consistent with the picture of phonon‐coupled electronic states predicted for fluorite oxides. Their confinement drives p‐type mixed conduction and enhances electromechanical coupling at room temperature.

This framework situates polarons in YSZ as the ionic analogue of color centres in wide‐band‐gap materials, where the degree of localization and lattice relaxation dictate transport and functionality. By tuning defect chemistry and orientation, we demonstrate that a classical ionic conductor can be transformed into a system with coexisting ionic, electronic, and electromechanical responses.

Overall, this study provides experimental validation for the quantum‐localization model of defects in fluorite oxides, establishing polaronic confinement as a general mechanism for engineering functional responses in ionic materials.

## 4 Methods

### 4.1 Pulser Laser Deposition

To systematically study the structural and electrochemical properties of YSZ films with different orientations, i.e., YSZ (100) and YSZ (111), films with various thicknesses ranging from 20 to 250 nm were deposited on different electrically insulating substrates, including NGO (111) and NGO (110), using the pulsed laser deposition (PLD) method. The YSZ films were deposited at a constant temperature of 650°C with the oxygen partial pressure in the chamber fixed to 10^−2^ mbar. Furthermore, to examine the effect of the underlying substrate on film quality, YSZ films were also deposited on Al_2_O_3_ (0001) under the same deposition conditions. All the films were deposited using a KrF excimer laser (248 nm wavelength, 25 ns pulse width). The laser beam was focused in a high‐vacuum chamber onto a target, with an energy density per pulse of about 3 J/cm^2^.

### 4.2 X‐Ray Diffraction

The crystallographic properties of the samples were investigated by XRD, XRR, and RSM using a Rigaku diffractometer SmartLab triple‐axis diffractometer with a rotating Cu anode as a source, operating at 200 mA and 50 kV, and using a one‐dimensional multi‐layered optic to collimate and mono‐chromatize the X‐ray beam point‐source, λ = 1.5418 Å.

### 4.3 Transmission Electron Microscopy

Electron‐transparent lamellas for transmission electron microscopy (TEM) analysis were prepared using a FEI Helios Nanolab 650 focused ion beam (FIB) microscope. Ir coating was applied for lamella preparation. High‐resolution transmission electron microscopy (HR‐TEM) was conducted on an FEI Talos F200X instrument, and the images were analyzed using Digital Micrograph software from Gatan.

### 4.4 Electrochemical Characterization

Electrochemical impedance spectroscopy (EIS) was performed using a frequency response analyzer connected to a dielectric interface (FRA, Solartron 1255 and 1290). The EIS measurements were conducted over a frequency range of 50 Hz to 1 MHz after applying symmetric electrodes in a two‐electrode configuration on the films using silver paste, with a lateral distance of approximately 0.8 mm. The EIS measurements were performed on the samples under different oxygen partial pressures (1 – 1 × 10^−30^ atm) and temperatures (450–750°C) to study the conductivity of YSZ films using Arrhenius and Brouwer plots.

### 4.5 Electromechanical Characterization

The electromechanical properties were characterized using a *Nano Vibration Analyzer* (SIOS) coupled with a signal generator and voltage amplifier to apply the electric field. The displacement signal was processed by a Lock‐in Amplifier (*Signal Recovery 7230*, Ametek), enhancing the signal‐to‐noise ratio and isolating a single‐frequency response. Because of the lock‐in amplifier, we do not differentiate between positive or negative displacement; thus, all reported stresses and coefficients are absolute values. Gold electrodes (30 nm thick) were deposited by sputtering in an in‐plane geometry with a 250 µm gap to apply the electric field. The sample was affixed in a cantilever geometry, and the generated stress was calculated using Stoney's formalism. Further details on the model and equations used to convert cantilever movement to stress are provided in Figure S14 and previous publications [52].

### 4.6 Ab‐Initio Calculations

Density Functional Theory (DFT) calculations have been used to investigate the positions of the Y‐doping and oxygen vacancies in the two YSZ films oriented 111 and 100. The calculations have been performed using the VASP package [53, 54] and the Atomistic Simulation Environment (ASE) [55]. All structures have been calculated using the PBEsol exchange‐correlation functional [56], adding a Hubbard correction of 3 eV on the Y and Zr atoms [57], the recommended pseudopotentials, an energy cutoff of 520 eV, and a *Gamma‐centered k‐point mesh of 4x4x4*. Moreover, dipole corrections were applied to the slab calculations. Once the bulk structure of ZrO_2_ has been fully optimized (forces < 0.05 eV/Å), we create two slab models with terminations (111) and (100) using a 2x2 supercell with 4 layers in the z‐direction. The slabs were separated by approximately 21 Å of vacuum to avoid interactions between periodic images. The YSZ(100) and YSZ(111) surfaces were modeled with lattice parameters of *a* = 7.283 Å, *b* = 7.283 Å, *c* = 30.123 Å, and angles α = β = 90°, γ = 120°. The bottom layers were fixed to mimic bulk‐like behavior, while the upper layers were fully relaxed. To determine the most stable surface configuration, several distributions of Y dopants and oxygen atoms were considered. Their relative free energies were compared, and the lowest‐energy configuration was selected for subsequent electronic‐structure analysis. To assess oxygen‐vacancy segregation, we compared the free energies of slab models containing oxygen vacancies placed at different depths (surface and subsurface positions), as well as the oxygen formation energies. The calculations show that configurations with vacancies located closer to the surface are energetically more favorable than those with vacancies in subsurface layers, as shown in Figure S15 and Table S3. The partial electronic charge density, electronic wave functions, and related post processings were obtained for the top of both valence and conduction bands using VASPKIT software [58]. The crystal structures used in the simulations were visualized using the VESTA software [59]. The atomic charges were determined using a Bader charge analysis, employing the grid‐based algorithm implemented by Henkelman et al. [60].

### 4.7 XPS and XAS

In situ X‐ray photoemission and X‐ray absorption spectroscopy (XPS and XAS) measurements were performed at APE‐HE beamline at Elettra synchrotron in Trieste. The samples were grown using the PLD system at Elettra with the same growth recipe optimized at DTU, and then transferred in situ from the PLD system to the analysis chamber without exposing the samples to air [61]. The in situ transfer procedure allows all samples to be measured under identical conditions, thereby limiting the effects of surface contaminants. Therefore, a reliable comparison of the spectroscopic properties of samples grown on different substrates and with different thicknesses is required. XPS measurements of Zr 3d, Y 3d, and O 1s core levels, as well as valence band (VB), were recorded with an Omicron EA125 hemispherical electron energy analyzer, with the sample at 45° with respect to the impinging linearly polarized light and normal to the surface. The XPS binding energy scale was calibrated using the adventitious carbon C 1s peak at 28X.X eV. XPS depth profiling was performed with a Kratos Axis Ultra using a 5 keV Ar‐ion beam. XAS measurements were performed at the Zr M_2,3_ absorption edges, corresponding to transitions from the 3p core states of Zr to conduction‐band states derived from 4d Zr atomic states. Data were acquired in total electron yield (TEY) mode at room temperature, normalizing the sample current to the incident photon flux at each energy in horizontal polarization with an incident angle of 45°. The valence band was also measured in resonance by using incident photons with the energy at the Zr M_3_ absorption edge.

### 4.8 UPS MAX IV

Ex situ ultraviolet photoelectron spectroscopy (UPS) was performed on two samples to investigate their electronic structures. The samples were electrically connected and measured using a hemispherical electron analyzer (SPECS Phoibos 150 R7 equipped with a 2D delay‐line detector). The Fermi level was referenced by measuring the tantalum clamp on the sample station. The work function difference between the two samples was determined by analyzing the secondary electron cutoff energy, with both samples biased at −7.23 V during measurement.

## Conflicts of Interest

The authors declare no conflicts of interest.

## Supporting information




**Supporting File**: smll73040‐sup‐0001‐SuppMat.pdf.

## Data Availability

Data will be made public via DOI dataset repository where the paper is accepted for publication.
