# Supplementary material for: Hole Polaronic Confinement in (111) Yttria‐Stabilised Zirconia
Source: Small. 2026 Mar 12;22(25):e13940. doi: 10.1002/smll.202513940 (PMC13137227; doi:10.1002/smll.202513940)
Supplement: Supplementary file 1 — Supporting File: smll73040‐sup‐0001‐SuppMat.pdf. [file SMLL-22-e13940-s001.docx]

**Supplementary Information**

**Hole polaronic confinement in (111) yttria-stabilised zirconia**

Milica Vasiljevic^1*^, Victor Buratto Tinti^1^, Javier Zamudio-García^1^, Jose Maria Castillo Robles^1^, Vasileios Bilalis^1^, Imran Asghar^1,2^, Simone Santucci^1^, Yichen Wu^1^, Simone Sanna^3,4^, Carmela Aruta^5^, Pasquale Orgiani^4^, Dimitris Koukolis^1^, David Marrero-López ^6^, Weimin Wang^7^, Ivano E. Castelli^1^, Vincenzo Esposito^1^*

^1^ DTU Energy, Technical University of Denmark, Fysikvej, Building 310, 2800 Kgs. Lyngby, Denmark

^2^ Renewable Energy Technologies Group, Faculty of Engineering and Natural Sciences, Tampere University, FI-33014, Finland

^3^ Universita' Degli Studi Di Roma Tor Vergata and CNR-SPIN Rome

Department of Civil Engineering and Computer Science, Via del Politecnico - 1, I - 00133, Roma, Italy.

^4.^ CNR-SPIN, via del Fosso del Cavaliere 100, Roma 00133, Italy 5. CNR-IOM Istituto Officina dei Materiali, I-34139 Trieste, Italy

^5^ CNR-SPIN, UOS Roma, Area della Ricerca di Tor Vergata, I-00133 Rome, Italy

^6^ Universidad de Málaga, Dpto. de Física Aplicada I, 29071-Málaga, Spain

^7^ Inversity of Lund, MAX IV Science Division, Lund, Sweden


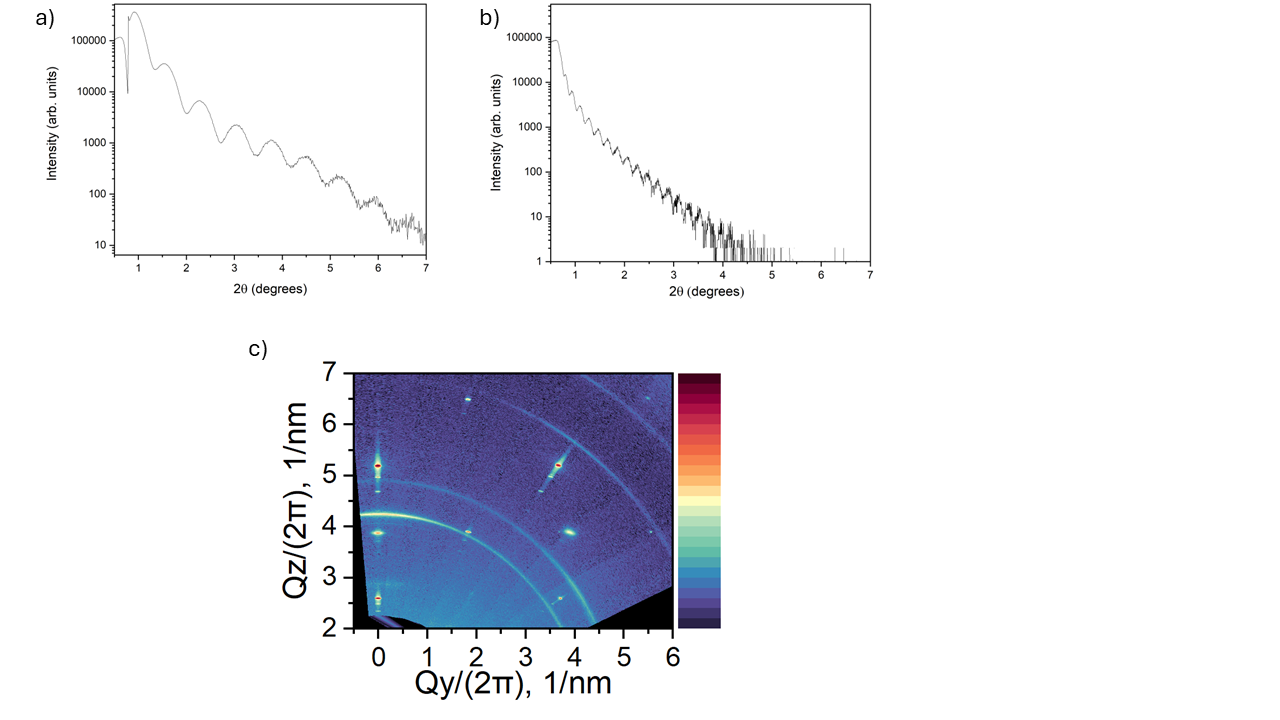


**Figure S1.** XRR measurement of a) thin YSZ (111) on NGO (100) and b) intermediate YSZ (100) film on NGO (110) single crystal substrate. c) Reciprocal space map of thin YSZ (100) film on NGO (110) substrate.


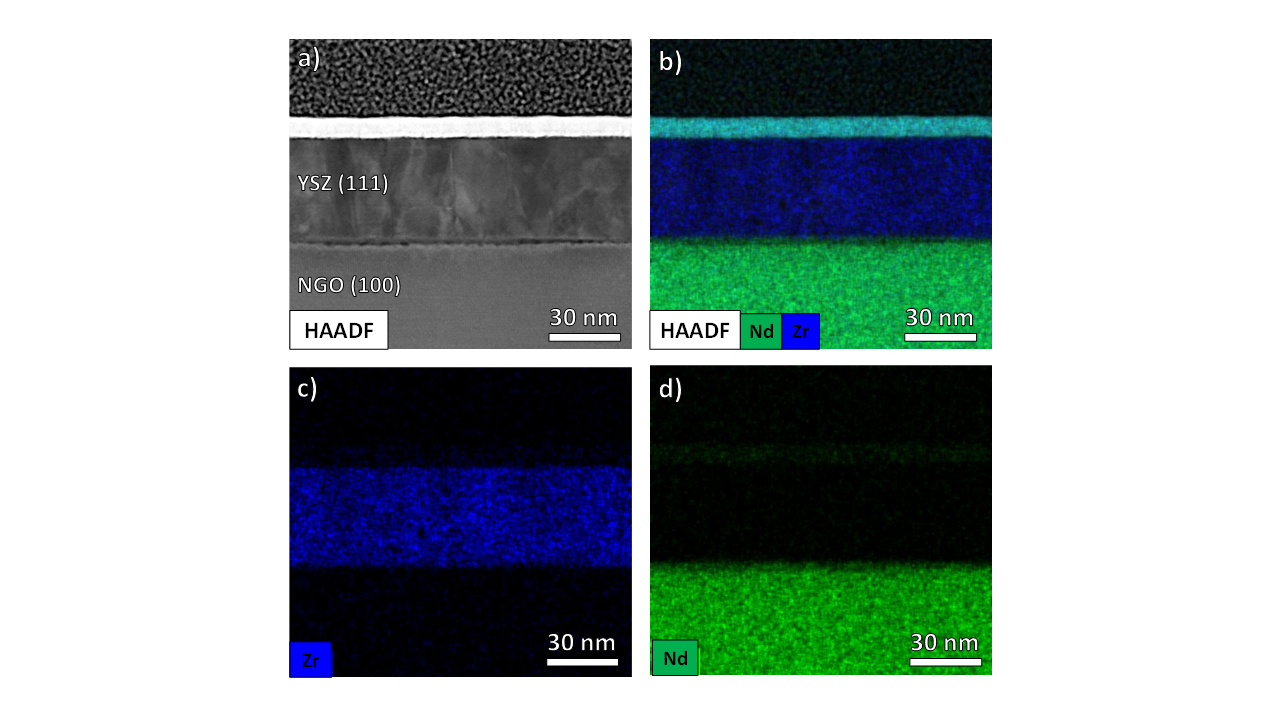


**Figure S2**: a-b) HAADF-STEM image of the intermediate YSZ (111) film deposited on NGO (100) single crystal substrate and c-d) EDX color mappings.


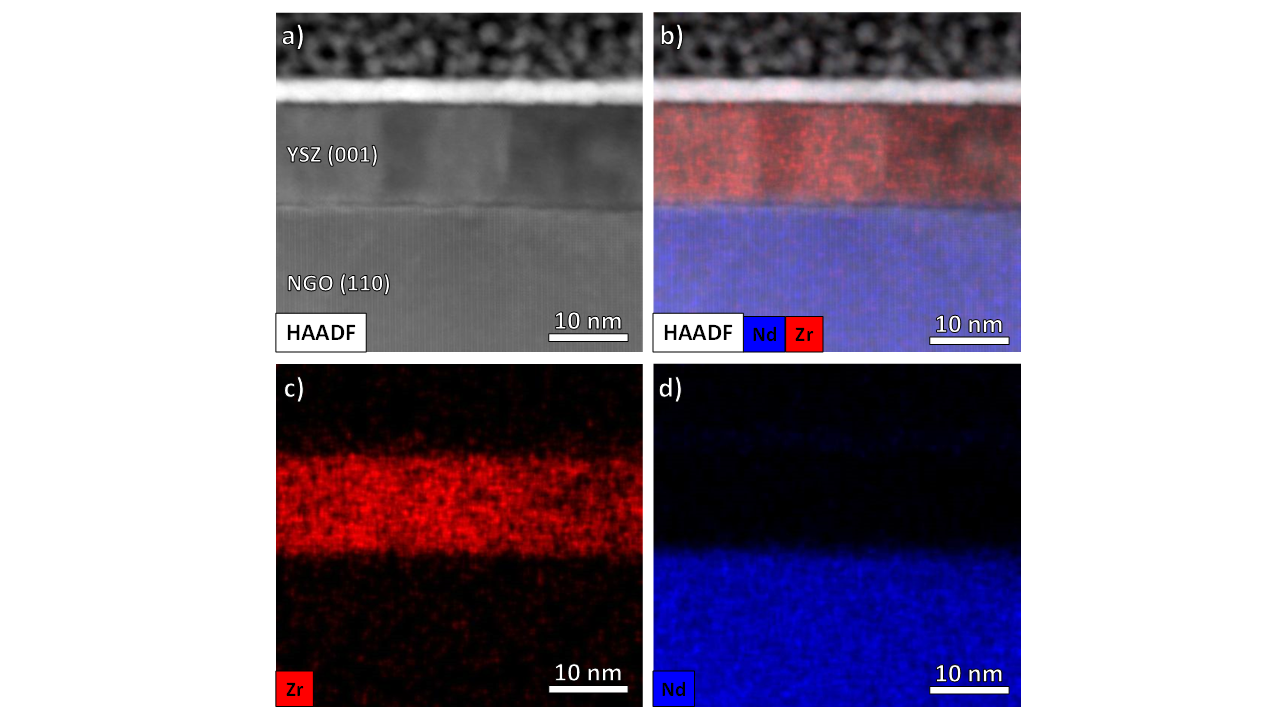


**Figure S3**: a-b) HAADF-STEM image of the thin YSZ (100) film deposited on NGO (110) single crystal substrate. and c-d) EDX color mappings.


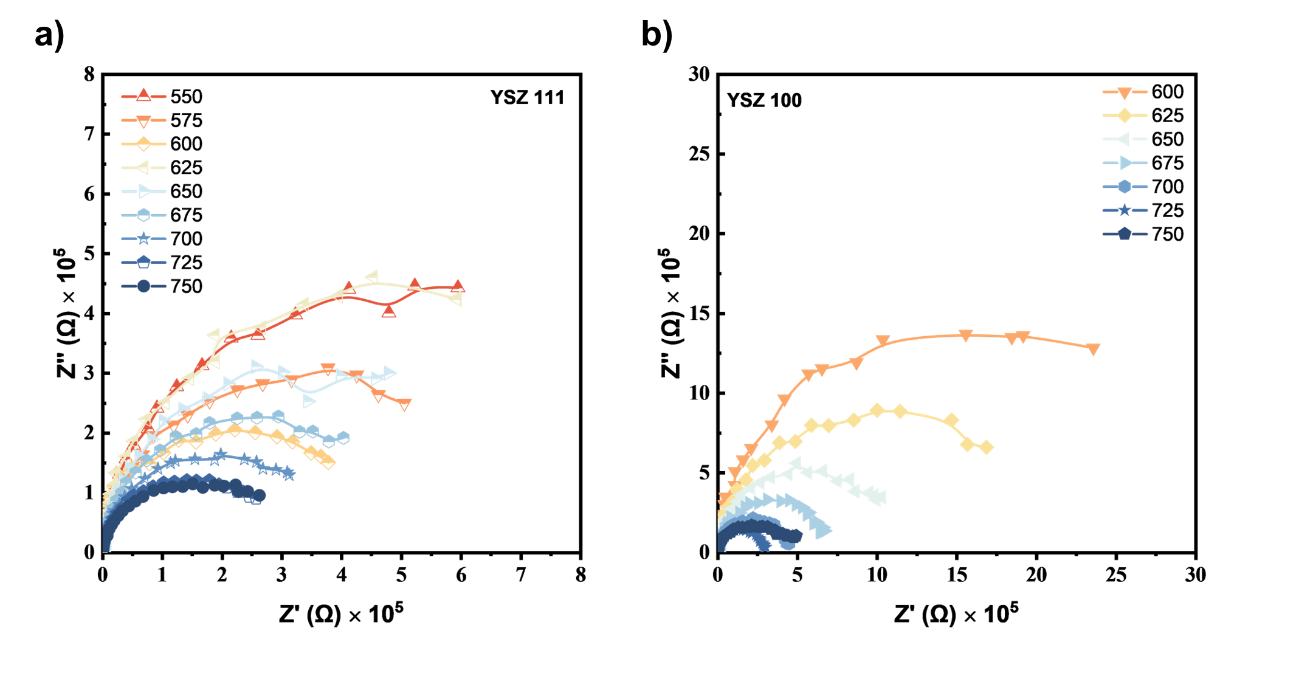


**Figure S4**: Nyquist plots obtained at different temperatures at pO_2_=0.21 atm for: a) YSZ (111) and b) YSZ (100) oriented sample.


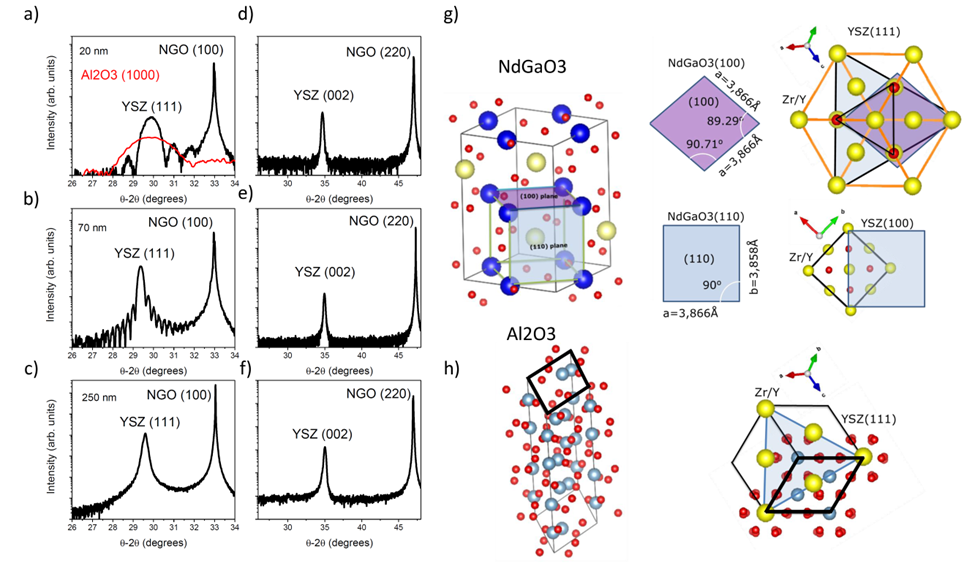


**Figure S5:** Schematic structure of the YSZ thin film on sapphire.

**Figure S6:** Nyquist plots of the YSZ (111) thin film sample with Pt blocking electrodes measured under three different gas atmospheres: oxygen (O₂), nitrogen (N₂), and 5% H₂ in Ar. The measurements demonstrate the impact of atmospheric composition on the impedance response, highlighting changes in the electrochemical behavior under oxidizing, inert, and reducing conditions. All data were collected at 625 ^o^C.


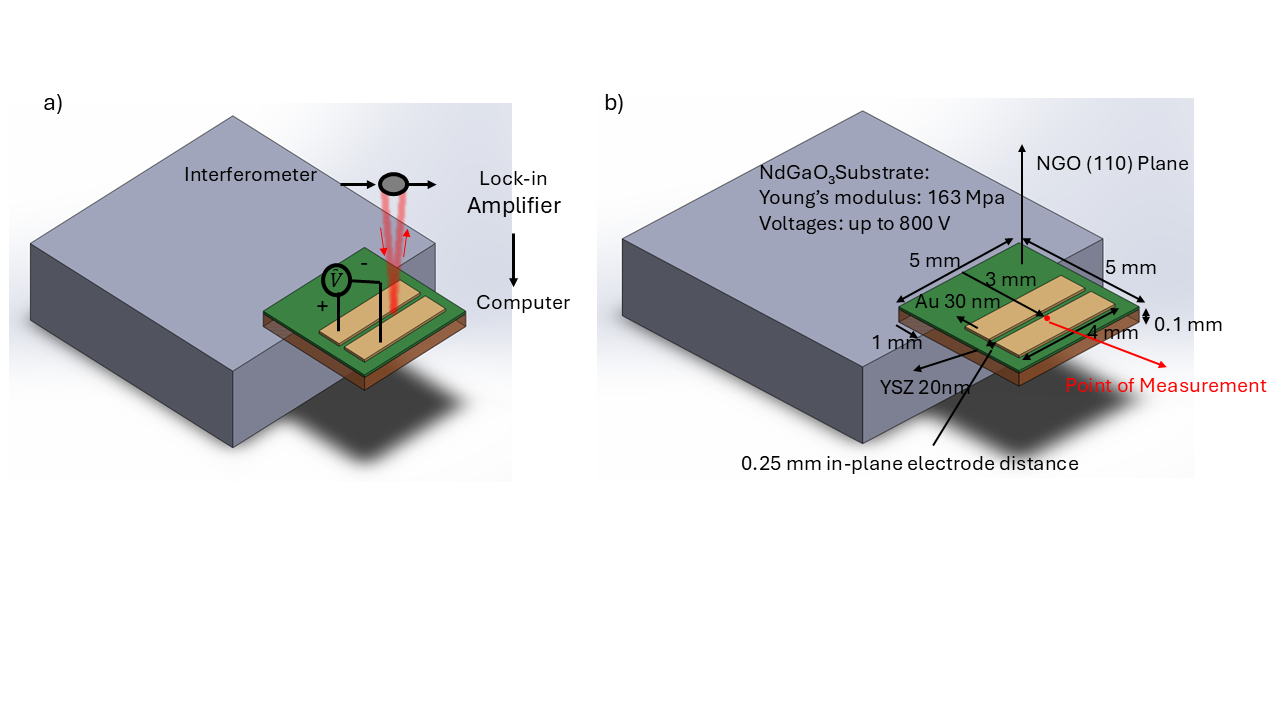


**Figure S7:** Schematic of a) the experimental setup and b) detailed sample configuration used in electromechanical measurements.

**Table S1**: Quantitative error values corresponding to the stress values of YSZ (111)/NGO (100) sample presented in **Figure 2d**. Errors are not visible in the plot due to their small magnitude relative to the symbol size.

| E^2^ (kV^2^/cm^2^) | Stress (MPa) | Error (MPa) |
| --- | --- | --- |
| 16 | 2.16661 | 0.14529 |
| 64 | 9.48566 | 0.17294 |
| 144 | 21.804 | 0.2113 |
| 256 | 39.75013 | 0.22143 |
| 400 | 61.99095 | 0.31054 |
| 576 | 92.20823 | 0.57112 |
| 784 | 126.59711 | 0.18817 |
| 1024 | 166.9718 | 0.97833 |

**Table S2:** Quantitative error values corresponding to the stress values of YSZ (100)/NGO (110) sample presented in **Figure 2d**. Errors are not visible in the plot due to their small magnitude relative to the symbol size.

| E^2^ (kV^2^/cm^2^) | Stress (MPa) | Error (MPa) |
| --- | --- | --- |
| 16 | -0.16037 | 0.15744 |
| 64 | 0.24657 | 0.21672 |
| 144 | 1.06802 | 0.19242 |
| 256 | 2.386 | 0.25075 |
| 400 | 3.8856 | 0.16181 |
| 576 | 5.76523 | 0.16238 |
| 784 | 7.8369 | 0.17358 |
| 1024 | 10.29508 | 0.20194 |

***
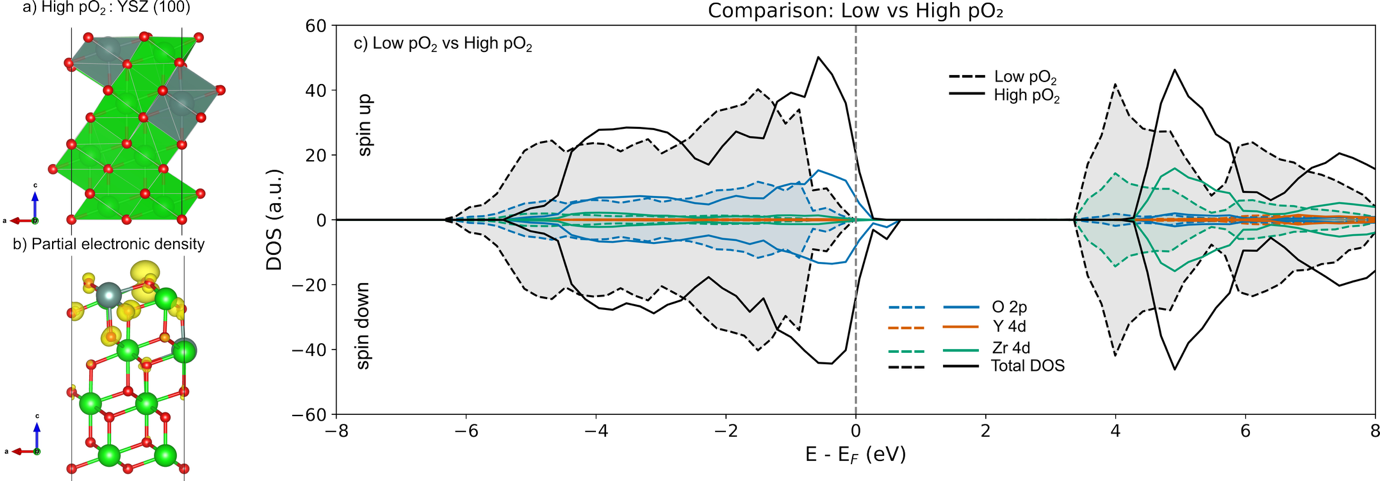
***

**Figure S8**: Schematic representation of the YSZ high pO_2_ structure used in the DFT calculations, the electronic charge density and the density of the states (DOS). The electronic charge density, represented in yellow, was calculated using an isosurface of 0.0008.


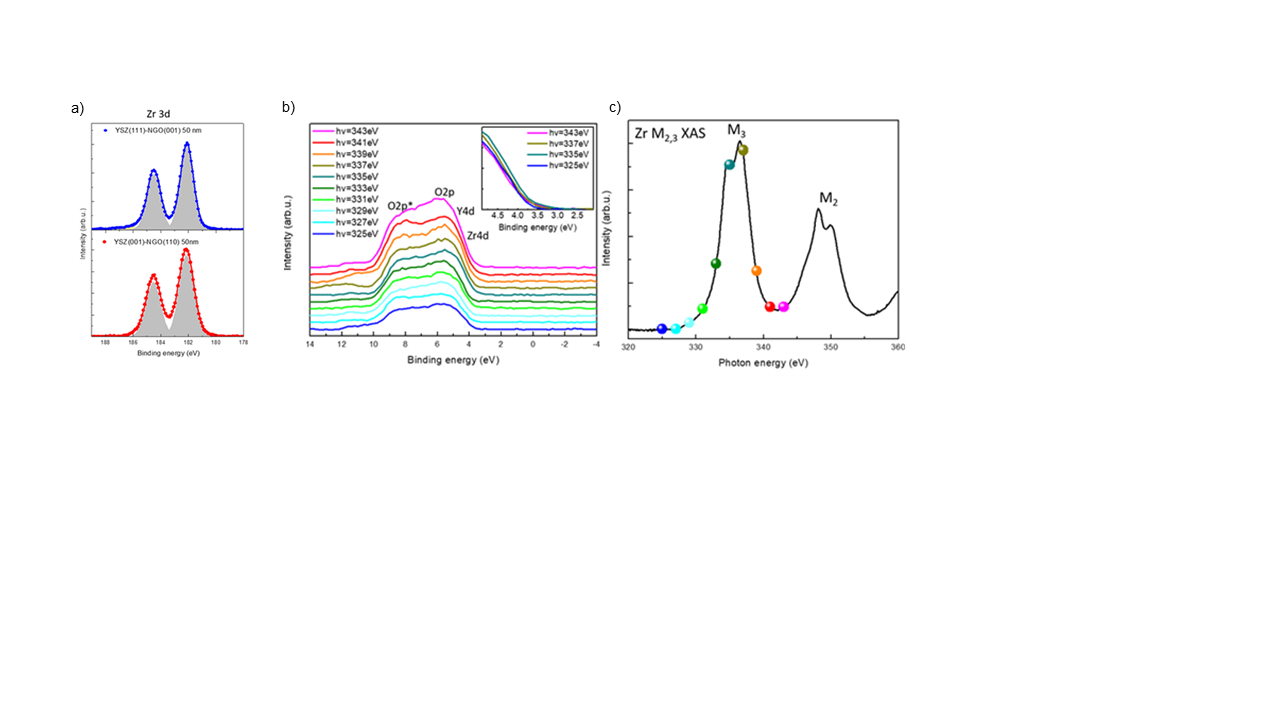


**Figure S9**: a) XPS spectra of 50 nm thick YSZ (111) and YSZ (100) films on NGO (001) and NGO (110), respectively, highlighting Zr 3d. Resonant photoemission electron spectroscopy measurements on YSZ (111) film grown on an Nb:STO (100) substrate. VB results are shown b) after normalization by the area at different photon energies scanning across the XAS Zr M3 - edge shown in c).


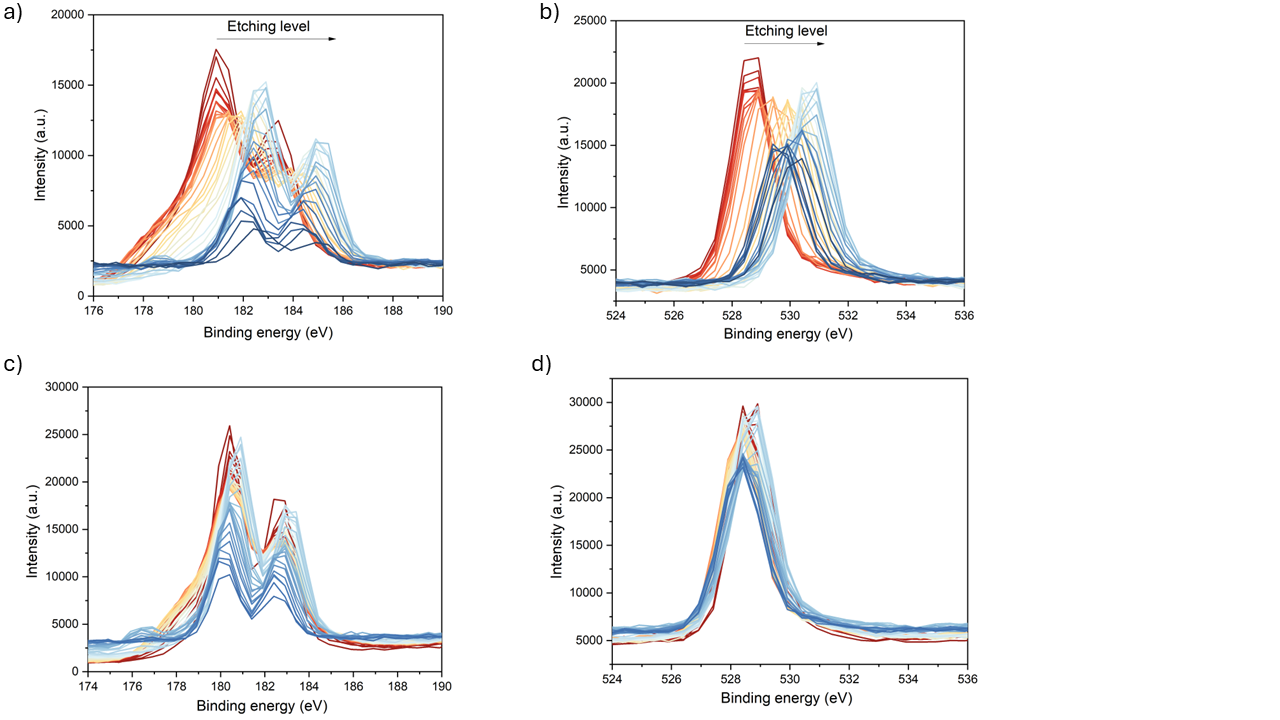


**Figure S10:** In-depth XPS spectra and highlighted shift of: a) Zr 3d and b) O1s in thin YSZ (111) and YSZ (100) films: c) Zr 3d and d) O1s. Experimental details are given in *Methods*.


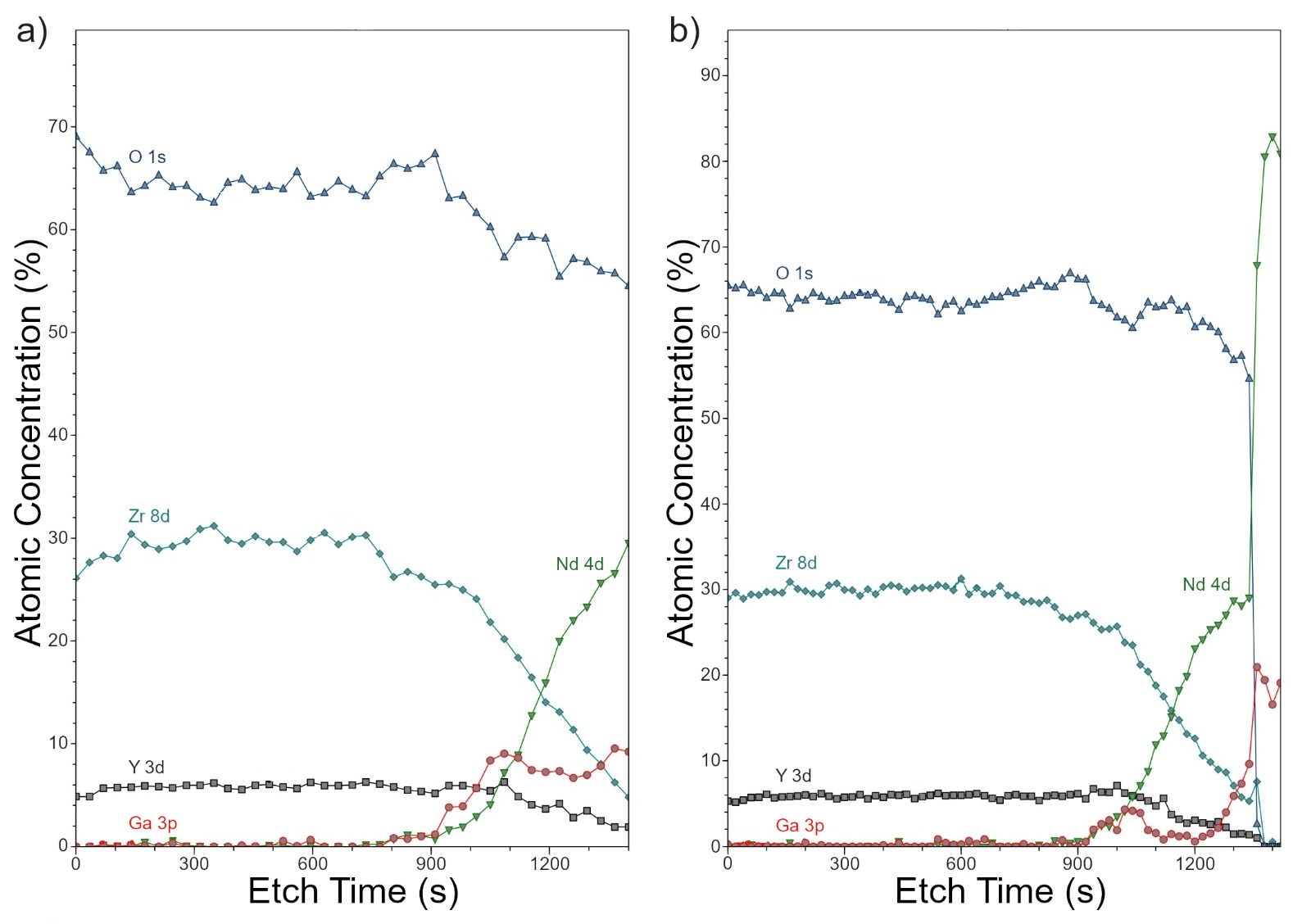


**Figure S11**: Atomic concentration calculated using in-depth XPS profiles in a) YSZ (111) and b) YSZ (100) sample.


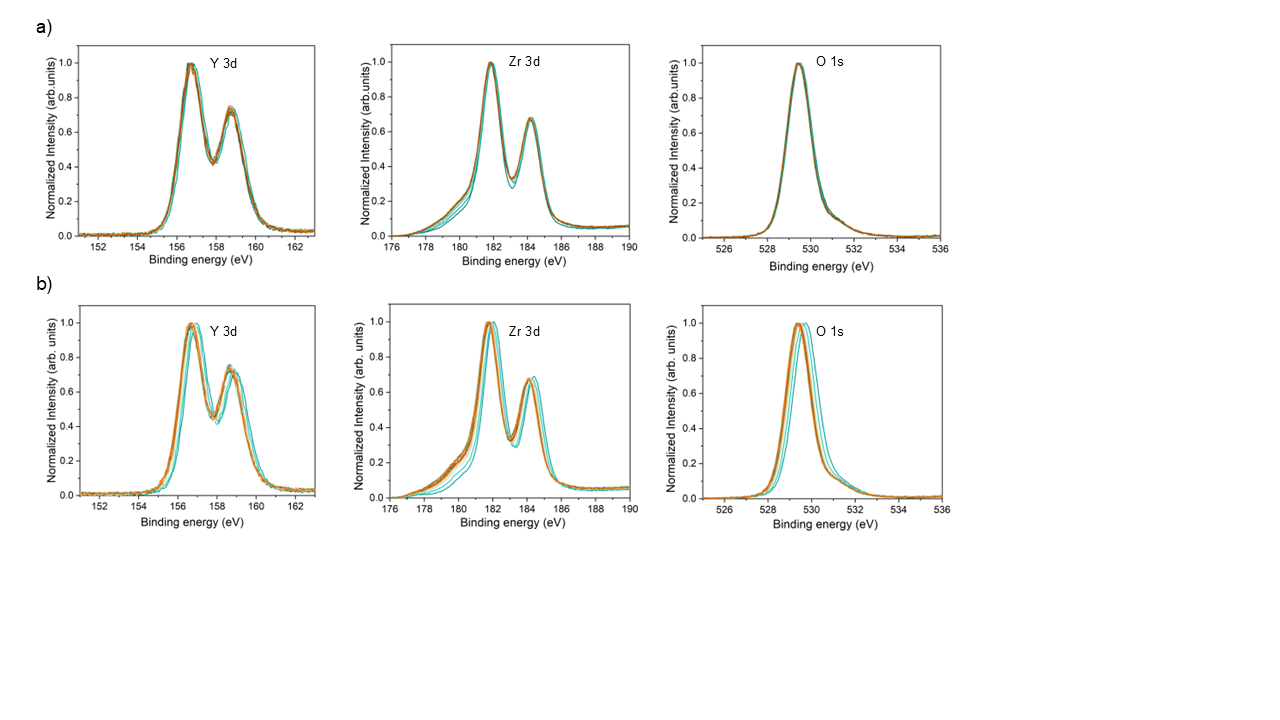


**Figure S12:** In situ XPS analysis of 70 nm thick a) YSZ (111) and b) YSZ (100) films showing uniform binding energies and elemental composition across the probed depth. The absence of significant shifts or gradients indicates chemical homogeneity throughout the film.


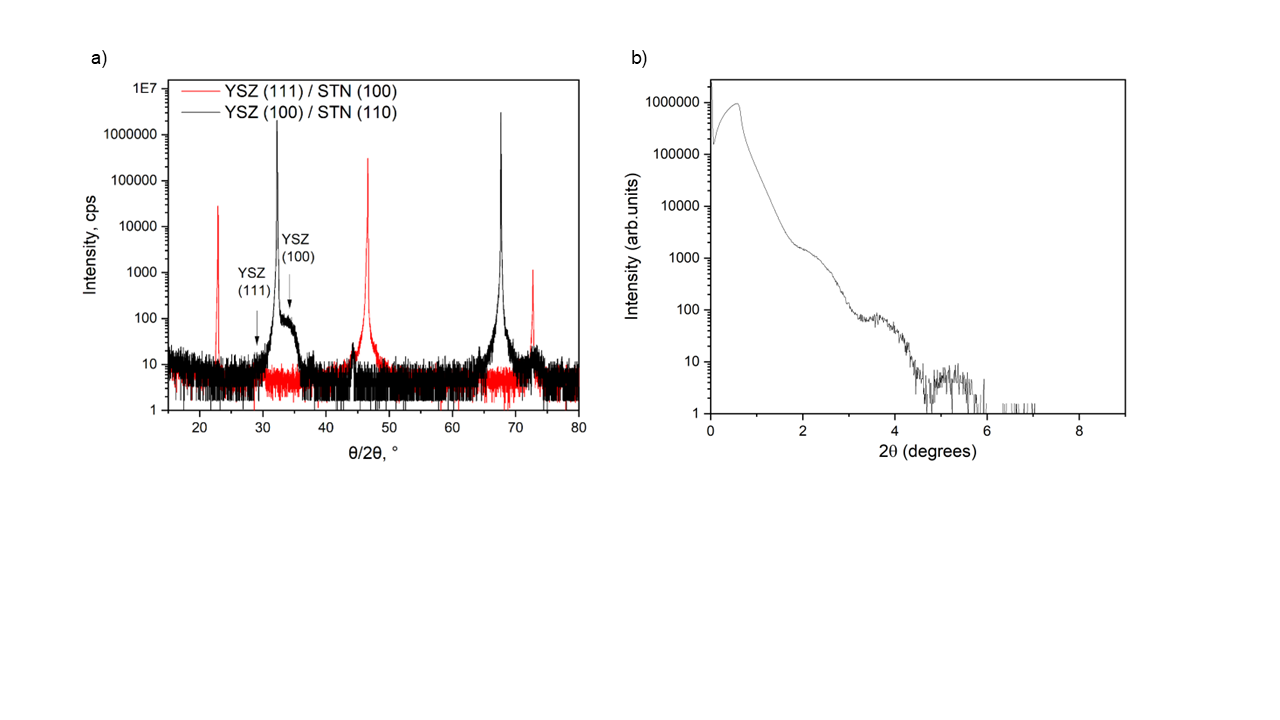


**Figure S13:** a) X-ray diffraction (XRD) patterns of thin YSZ (111) and (100) films deposited on Nb:STO single-crystal substrates with (110) and (100) orientations, respectively, confirming epitaxial growth and phase purity. b) X-ray reflectivity (XRR) measurement of the YSZ (111) film, indicating a thickness of approximately 4 nm. This ultrathin geometry was selected to meet the spatial resolution requirements of the technique, enabling clear observation of interfacial features between the film and substrate.


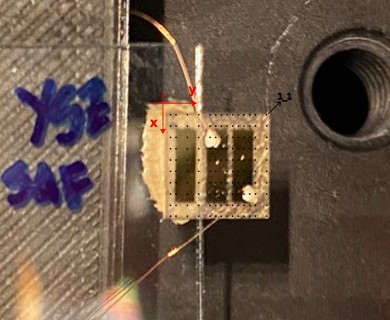

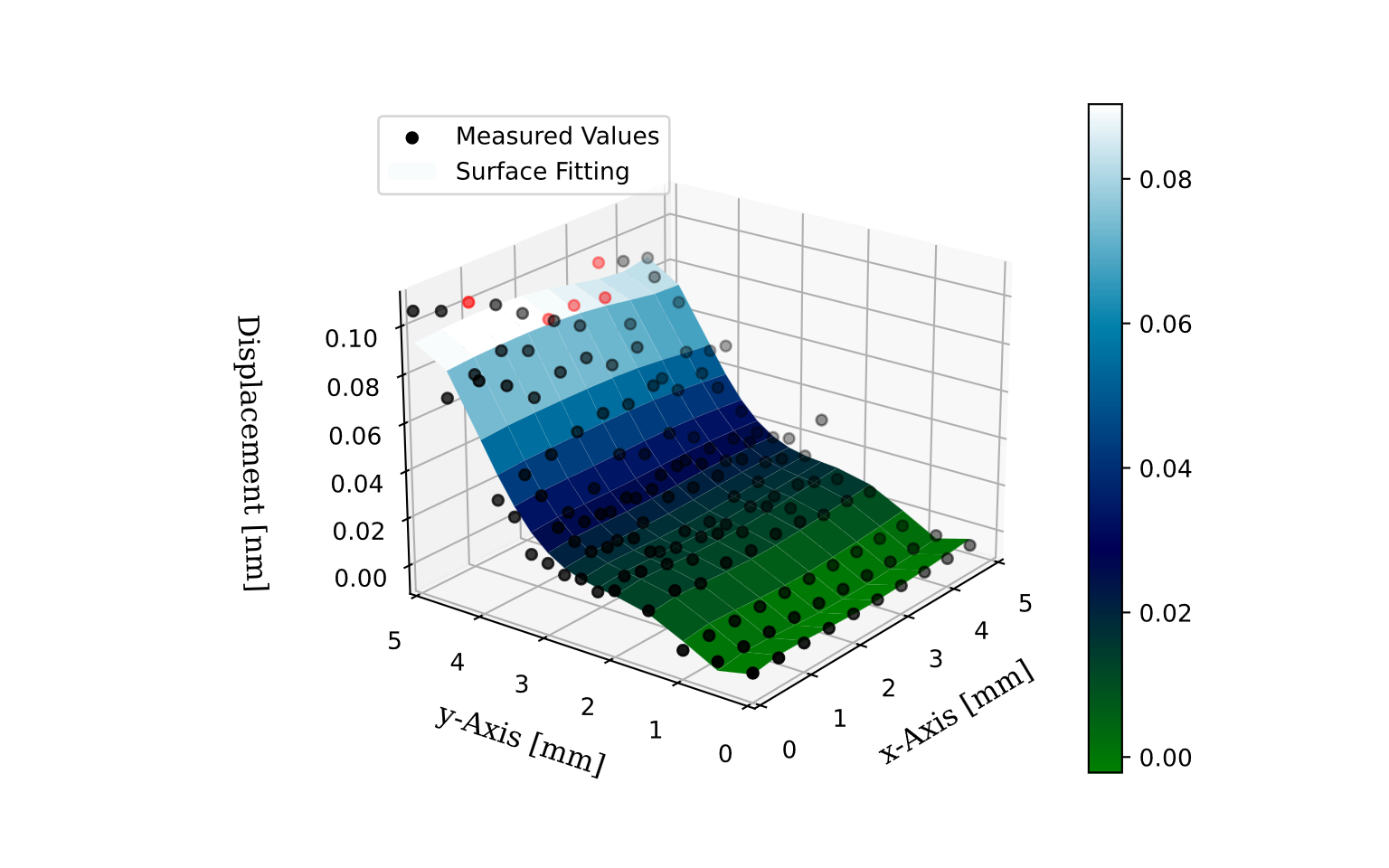


**Figure S14**: (left) Picture of the sample configuration under the laser interferometry; (right) displacement mapping over different points in the surface for the YSZ (111) on sapphire at 10 Hz and 800 V. Red points are averaged due to high signal-to-noise ratio. The displacement of the surface (2^nd^ harmonic) of the YSZ (111) on the sapphire sample was mapped under an applied sinusoidal wave of 800V, 10Hz. Polynomial of 6^th^ order was used to fit the measured values. The noise of the measurements was around 3.5uV and was subtracted from the values.


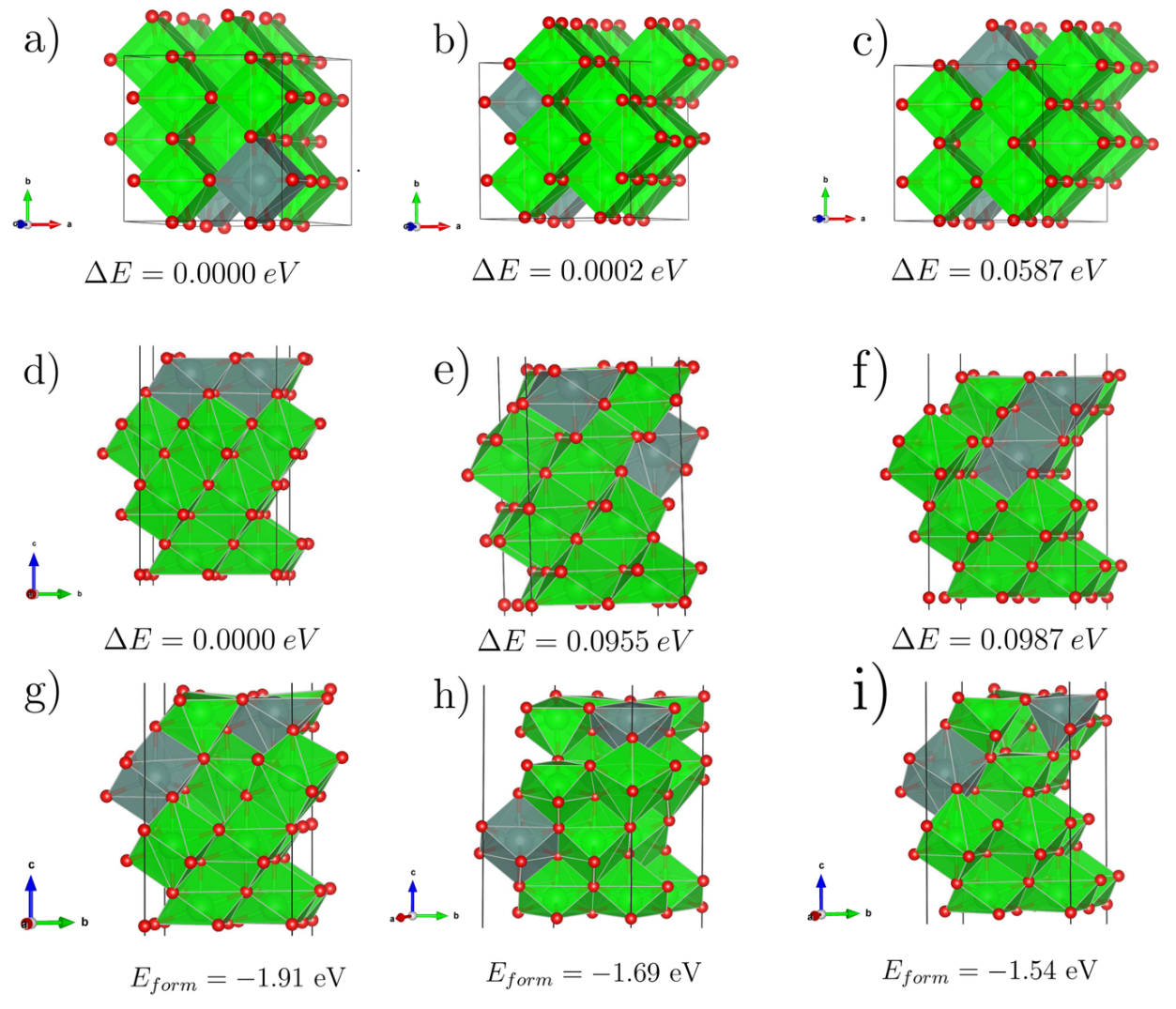


**Figure S15**: Most stable structures for the YSZ system: **(a–c)** the bulk supercell, **(d–f)** the pristine YSZ(111) surface slab, and **(g–i)** the defective YSZ(100) surface slab containing an oxygen vacancy. Green, gray, and red spheres represent Zr, Y, and O atoms, respectively. For the supercell and the YSZ(100) slab, the most stable dopant configurations were determined by means of the relative energy, calculated as $\begin{aligned} \Delta E=E_{tot}^{config}-E_{tot}^{min} \end{aligned}$, where $\begin{aligned} E_{tot}^{config} \end{aligned}$is the total DFT free energy of the specific configuration being evaluated and $E_{tot}^{min}$ is the total DFT free energy of the most stable configuration found in that structural set. For the YSZ(111) models, the stability as a function of depth was determined by means of the oxygen vacancy formation energy, calculated as $\begin{aligned} E_{form}=E_{defect}-E_{pristine}+E_{O} \end{aligned}$, where $E_{defect}$ is the total energy of the slab containing the oxygen vacancy, $E_{pristine}$ is the total energy of the corresponding perfect (defect-free) slab model, and $E_{O}$ is the reference energy of a single oxygen atom.

**Table S3**: Calculated oxygen vacancy formation energies E_form_ by depth for the most favourable YSZ(100) slabs. The energetic minimum at the subsurface (Layer 5) confirms thermodynamic vacancy segregation.

| Slab Model | Layer 6 | Layer 5 | Layer 4 | Layer 2/ Layer 3 |
| --- | --- | --- | --- | --- |
| **2Y-ZrO2_slab_12** | -1.35 | -1.91, 1.54 | -1.45 | -0.21 |
| **2Y-ZrO2_slab_14** | -0.86 | -1.69 | -1.22 | -0.85 |
| **2Y-ZrO2_slab_13** | -0.64 | -1.38 | -1.41 | -- |
| **2Y-ZrO2_slab_4** | -0.60 | -1.34 | -1.37 | -- |
